# The Gap Detection Test: Can It Be Used to Diagnose Tinnitus?

**DOI:** 10.1097/AUD.0000000000000156

**Published:** 2015-06-24

**Authors:** Kris Boyen, Deniz Başkent, Pim van Dijk

**Affiliations:** 1 Department of Otorhinolaryngology/Head and Neck Surgery, University of Groningen, University Medical Center Groningen, Groningen, The Netherlands; 2 Graduate School of Medical Sciences, Research School of Behavioural and Cognitive Neurosciences, University of Groningen, Groningen, The Netherlands; and 3 Department of Speech and Language Therapy, Hanze University Groningen, University of Applied Sciences, Groningen, The Netherlands.

**Keywords:** Diagnostic tool, Gap detection threshold, Tinnitus

## Abstract

**Objectives:**

Animals with induced tinnitus showed difficulties in detecting silent gaps in sounds, suggesting that the tinnitus percept may be filling the gap. The main purpose of this study was to evaluate the applicability of this approach to detect tinnitus in human patients. The authors first hypothesized that gap detection would be impaired in patients with tinnitus, and second, that gap detection would be more impaired at frequencies close to the tinnitus frequency of the patient.

**Design:**

Twenty-two adults with bilateral tinnitus, 20 age-matched and hearing loss–matched subjects without tinnitus, and 10 young normal-hearing subjects participated in the study. To determine the characteristics of the tinnitus, subjects matched an external sound to their perceived tinnitus in pitch and loudness. To determine the minimum detectable gap, the gap threshold, an adaptive psychoacoustic test was performed three times by each subject. In this gap detection test, four different stimuli, with various frequencies and bandwidths, were presented at three intensity levels each.

**Results:**

Similar to previous reports of gap detection, increasing sensation level yielded shorter gap thresholds for all stimuli in all groups. Interestingly, the tinnitus group did not display elevated gap thresholds in any of the four stimuli. Moreover, visual inspection of the data revealed no relation between gap detection performance and perceived tinnitus pitch.

**Conclusions:**

These findings show that tinnitus in humans has no effect on the ability to detect gaps in auditory stimuli. Thus, the testing procedure in its present form is not suitable for clinical detection of tinnitus in humans.

## Abstract 2

Animal experiments suggest that tinnitus may be related to difficulties in the ability to detect silent gaps in sound. To test this, 22 subjects with hearing loss and bilateral continuous tinnitus, 20 age- and hearing-loss-matched subjects without tinnitus, and ten young normal-hearing controls performed a gap detection test. The authors could not prove evidence for a difference in performance between the subjects with and without tinnitus, leading to the conclusion that the testing procedure in its present form is not suitable for detecting tinnitus in humans.

## INTRODUCTION

Tinnitus is a poorly understood auditory percept that occurs in the absence of an external stimulus and is typically associated with hearing loss. It is a common disorder with prevalence estimates ranging from 7 to 20% among randomly selected populations (Hoffman & Reed 2004). Most patients with chronic tinnitus are able to cope effectively with the disturbance, despite being continuously aware of the tinnitus percept. However, for some patients, the tinnitus is more than a trivial annoyance and can result in feelings of desperation (Dobie 2003).

Investigations of the perceptual characteristics of tinnitus and of how these characteristics relate to other auditory variables are required to further understand the underlying generating mechanisms of tinnitus (Tyler 1991). Currently, evaluation of tinnitus greatly relies on subjective measures, such as questionnaires, visual rating scales, and other self-reports (for a review, see Holgers et al. 2003). The perceptual characteristics of tinnitus are usually assessed by a matching procedure, where loudness and pitch of an external sound are matched to those of the tinnitus percept (Tyler 2000; for a review, see Holgers et al. 2003). These matching methods generate variable results that are not well replicated from one measurement to another, even in the same exact patient (Penner 1983; Tyler & Conrad-Armes 1983; Penner & Bilger 1992). Therefore, a more robust measure of tinnitus is highly desirable.

Neuroscientists, who use animal models in the study of tinnitus, have developed behavioral paradigms to assess tinnitus in laboratory animals. In the initial classic paper by Jastreboff et al. (1988), rats were trained to stop licking a water supply when a 30-sec silent period was introduced in an ongoing stimulus. Half of the animals were treated with salicylate, which is known to cause tinnitus in humans (Cazals 2000). In the treated animals, the 30-sec pause caused only partial suppression of the licking behavior. This behavioral response, expressed in a suppression ratio, was interpreted to imply the perception of tinnitus in the treated animals: the animals no longer experience silence, presumably due to the tinnitus percept filling the silence. A modified paradigm was used by Bauer and Brozoski (2001), who used noise trauma to induce tinnitus. These authors also addressed an important issue: Could the abnormal behavior be related to hearing loss that is caused by the noise trauma? To assess this potential factor, the authors tested rats with unilateral trauma and thus made sure that the animals would hear the silent period with the untraumatized ear. Bauer and Brozoski thus excluded the possibility that peripheral hearing loss due to the trauma caused the failure to respond to silence, leading to the conclusion that the reduced ability to detect the silence must have been caused by tinnitus.

Importantly, in the paradigms developed by Jastreboff et al. (1988) and Bauer and Brozoski (2001), the animals were trained to actively listen to their acoustic environment to be able to modify their behavior accordingly. In contrast, a new paradigm developed by Turner et al. (2006) explored the characteristics of an acoustic reflex as a correlate of tinnitus. In this new paradigm, the animal’s response is not based on learned behavior. Instead, the paradigm assesses suppression of the acoustic startle reflex (ASR). The ASR is a primitive response based on neural circuits in the lower brainstem, which is elicited by a sudden loud sound (Basavaraj & Yan 2012). It is possible to inhibit the ASR by presenting quieter sounds less than 500 msec before the onset of the startle stimulus (Hoffman & Searle 1965). This effect is called prepulse inhibition. As shown by Turner et al., a brief silent gap in ongoing noise also acts as a prepulse, inhibiting the ASR to a sudden loud noise (Ison 1982; Ison et al. 2005). Turner et al. made use of silent gaps in ongoing noise as a prepulse to inhibit the ASR in rats. The principle finding by Turner et al. is that animals that are exposed to loud traumatizing sound show poorer performance in detecting the gap. By performing control experiments, Turner et al. further showed that this poorer performance is most probably not caused by hearing loss. As in the studies by Jastreboff et al. (1988) and Bauer and Brozoski (2001), the failure to detect the silent gap is interpreted as evidence of tinnitus. Moreover, their results suggest that the decrease in gap detection is related to the presumed tinnitus pitch; such an effect was most strongly observed when the signal that contained the gap was most similar in spectral content to that of presumed tinnitus percept. In addition, a comparison of the performance of animals in the traditional silence detection task (Bauer & Brozoski 2001) and the new startle suppression paradigm (Turner et al. 2006) showed correlated results, suggesting that both paradigms may similarly detect tinnitus.

In contrast to many studies with animals, tinnitus studies on human subjects have been scarce. In 2013, Fournier and Hébert (2013) used a gap detection startle paradigm in humans with tinnitus. The startle response was recorded by measuring electromyography activity of the eye blink. The startle inhibition was determined in various conditions: using (1) only a startle stimulus, (2) a startle stimulus preceded by a prepulse, and (3) a startle stimulus preceded by a gap of 50 msec in ongoing noise. The ongoing noise was either a low-frequency (200–1200 Hz, centered at 500 Hz) or a high-frequency (3.5–4.5 kHz, centered at 4 kHz) noise. The results showed that patients with tinnitus had normal inhibition of the ASR when a startle stimulus was preceded by a brief prepulse but displayed significantly less inhibition when it was preceded by a silent gap in ongoing noise. The impaired inhibition was observed for the low-frequency and high-frequency ongoing noise although all participants had high-frequency tinnitus. Thus, there is no obvious relation between the pitch of the ongoing external stimulus that contained the gap and the tinnitus percept. Therefore, Fournier and Hébert (2013) concluded that tinnitus is not simply “filling in the gap.” However, these results suggest that gap detection or the startle reflex, or both, could be abnormal in patients with tinnitus. These results thus motivate the search for a reliable behavioral correlate of tinnitus in humans.

If suppression of the startle reflex is abnormal in tinnitus, it could be that tinnitus subjects simply do not perceive the silent gap. This hypothesis was recently investigated by Campolo et al. (2013). To match previous animal and human studies, gaps of 50-msec duration were used. Campolo et al. observed no deficit in detection of the gap. Based on these results, Campolo et al. suggested that tinnitus interferes with the gap prepulse inhibition of the ASR but not with perceiving the gap in general.

Alternatively, it may be that the detection of a 50-msec gap is just a too simple task for human subjects. Therefore, we set out to increase the difficulty of the gap detection task by measuring the smallest possible gaps that subjects with and without tinnitus can detect. Confirming a gap detection deficit in humans with tinnitus will provide important validation of the behavioral gap detection paradigms used in animal studies and may also provide guidelines for a potential novel diagnostic tool for patients with tinnitus. However, if gap detection is normal in human tinnitus subjects, it must be concluded that the behavioral gap detection paradigms used in animal studies cannot be translated to human applications in a straightforward way.

## MATERIALS AND METHODS

### Subjects

The study population consisted of three different groups of subjects. The main group of interest consisted of 22 subjects who perceived bilateral continuous tinnitus (Tin group). Gap detection is known to depend both on age (Snell & Frisina 2000; Roberts & Lister 2004; Pichora-Fuller et al. 2006) and hearing loss (Florentine & Buus 1984; Roberts & Lister 2004). To account for these effects, the second group, consisting of 20 subjects without tinnitus (NoTin group), served as a matched control group with similar age, gender, and hearing characteristics (Table 1). The subjects of both groups were recruited at the University Medical Center Groningen, The Netherlands, or via flyers. The third group consisted of 10 young normal-hearing subjects without tinnitus (Con group) and served as a reference group. This last group was recruited via flyers.

**TABLE 1.**
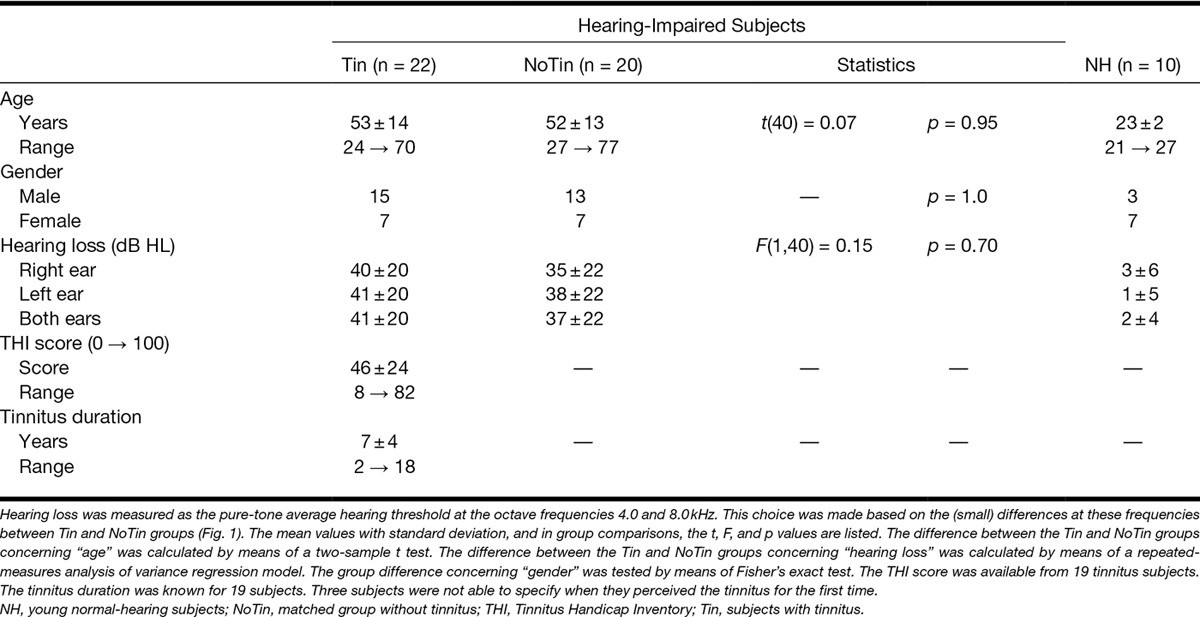
Subject characteristics

Pure-tone audiometry was performed in all subjects at six different octave frequencies (0.25, 0.5, 1, 2, 4, and 8 kHz) in each ear. To be enrolled in the study, all subjects had to have symmetric hearing levels. Threshold differences between ears were 20 dB or less for at least five of the six test frequencies. The subjects of the Con group additionally had to have hearing thresholds at all octave frequencies ≤20 dB HL. None of the subjects had a major medical, neurological, or psychiatric history.

**Fig. 1.**
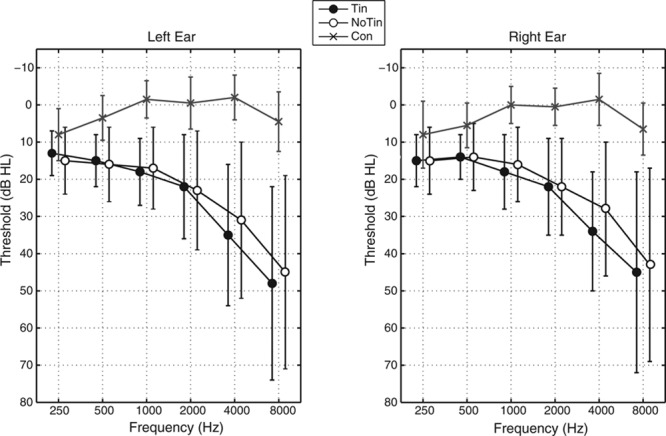
Mean audiograms for the Tin group, the NoTin group, and the Con group. The error bars indicate the group standard deviations around the mean. Con indicates young normal-hearing subjects; NoTin, matched group without tinnitus; Tin, tinnitus.

Details of participant characteristics are listed in Table 1. No significant differences in population characteristics between the Tin and NoTin groups were observed. Age was significantly correlated with hearing loss on both ears (*R* = 0.75, *p* < 0.001). The mean audiogram per group is shown in Figure 1. Clear differences between the Con group and both other groups are visible in the high-frequency range. The age difference between the Tin and NoTin groups was tested using a *t* test. The difference in gender was tested by Fisher’s exact test. Differences between the hearing thresholds were tested using a repeated-measures analysis of variance (ANOVA) regression model with group (Tin versus NoTin) as a between-subject factor and frequency (six octave frequencies) and ear (left versus right) as within-subjects factors. These statistical analyses did not show significant differences between the Tin and NoTin groups (Table 1).

This study was approved by the Medical Ethics Committee of the University Medical Center Groningen. All subjects gave written informed consent in accordance with Dutch legislation.

### Stimuli

The gap detection test was performed for four different band-passed (BP) stimuli of Gaussian noise at three different sound levels above threshold: 5 dB SL, 10 dB SL, and 25 dB SL, with dB SL as a unit relative to the hearing threshold of each subject. The gap detection test was preceded by a training session using a white Gaussian noise at 25 dB SL. All sounds were presented binaurally. The BP Gaussian noise test stimuli had frequency contents of 4000 to 8000 Hz (1-octave noise), 4000 to 5000 Hz (1/3-octave noise), 5000 to 6300 Hz (1/3-octave noise), and 6300 to 8000 Hz (1/3-octave noise). The stimuli were BP filtered using a Butterworth filter, with filter order of 1 and a corresponding filter slope of 6 dB/octave. The gap was produced by modulating the stimulus with a square wave. To prevent BP-filtering effects on the square wave, the gap was applied after the filtering. A raised cosine ramp of 6 msec was applied to the onsets and offsets of the square wave to prevent spectral splatter. All stimuli had a duration of 300 msec. In each trial, the onset gap was randomly placed within the stimulus between 100 and 200 msec from the onset. On a Macintosh computer, signal processing and stimulus presentation were performed in MATLAB R2012b (The Mathworks Inc., Natick, MA) with a sampling frequency of 48.0 kHz.

For calibration, the sound level of each stimulus was adjusted to 85 dB SPL. The calibrated sound level was subsequently modified to present the stimuli at the required sound levels. To provide the stimuli at sound levels as correct as possible, the hearing thresholds for all five stimuli (four test stimuli and one training stimulus) were binaurally determined before each test session, following the standard modified Hughson–Westlake procedure (Harrell 2002). The hearing threshold of each stimulus was first determined in steps of 5 dB SPL until the first estimated threshold was found; thereafter, the threshold measurement was repeated with step sizes of 2 dB SPL for better precision.

### Equipment

All testing was conducted in a sound-isolated booth. For calibration, a sound level meter (Svan 979; Svantek) and a Kemar (G.R.A.S.) head and torso simulator were used. The processed stimuli were sent as digital signals through the S/PDIF output of AudioFire 4, the external soundcard of Echo Digital Audio Corporation (CA). After conversion to an analog signal via DA10 digital-to-analog converter of Lavry Engineering Inc. (WA), they were presented binaurally to the subject with HD-600 headphones of Sennheiser Electronic Corporation (CT). The gap detection test was administered via a graphical user interface programmed in MATLAB R2012b (The Mathworks Inc.), which was shown on a computer screen in front of the subject.

### Procedure

For all subjects, the test session was repeated three times, spread over different days, but completed in a maximum of 70 days. To minimize the possible effect of fatigue, the Tin subjects were encouraged to have two breaks, each at least 5 min of duration, during each session; all other subjects had one break. Each participant was explicitly told that more breaks could be taken whenever necessary. Each session lasted for a maximum of two and a half hours including breaks, leading to a maximum testing duration of seven and a half hours per subject.

#### Tinnitus Characteristics

In subjects with tinnitus, the date when tinnitus was first perceived and, if available, the scores of the Tinnitus Handicap Inventory were documented from the medical files. In addition, all tinnitus subjects performed a tinnitus pitch and loudness matching task during each test session. In this matching task, the stimuli were presented to the right ear only. Initially, the subjects were asked to indicate whether their tinnitus most resembled a tone, a narrowband noise (1/3-octave), or a wideband (WB) noise. If a tone or narrowband noise was chosen to most closely resemble the tinnitus, the procedure was started with a corresponding sound of 1-kHz center frequency, presented at 10-dB above hearing threshold. This sound was adjusted in frequency, using a step size of 1/8th octave, until subjectively the best matching frequency was reached. Next, the sound level was adjusted in steps of 5 dB until the loudness matched the tinnitus loudness most closely. Starting from the resulting best matching sound, the frequency and loudness matching procedures were subsequently repeated with step sizes of 1/16th octave and 1 dB, respectively. If the subject matched the tinnitus with a WB noise, only the loudness match was performed. To express the intensity level of the matched sound in dB SL, the hearing threshold for the matched sound was measured. For the patients with tinnitus of whom results of a previous tinnitus matching task were available, the procedure was started at this frequency. Tinnitus matching frequencies were not measured for frequencies higher than 8 kHz. For subjects with a tinnitus frequency higher than 8 kHz, an 8-kHz sound was used for the loudness matching. All tinnitus subjects performed the tinnitus matching task before each of the three test sessions, except one subject who performed the matching task only twice.

To verify whether the gap detection test influenced the tinnitus loudness, tinnitus subjects rated tinnitus loudness on a numeric rating scale before and after each data collection session. The numeric rating scale ranged from 0 (tinnitus not audible at the time) to 10 (tinnitus sounds as loud as imaginable).

#### Gap Detection Test

For each subject, the gap detection thresholds (GDTs) for the four different test stimuli with varying frequencies and bandwidth (4000–8000, 4000–5000, 5000–6300, and 6300–8000 Hz), presented at three different intensity levels above their respective hearing thresholds (5, 10, and 25 dB SL), were tested in randomized order, leading to a total number of 12 runs per session. The test was preceded by a training session using a white Gaussian noise stimulus presented at 25 dB SL. When the sound intensity was higher than 80 dB SPL or when the subject reported the stimulus as too loud, the respective run was not performed or terminated. The specific parameters for the procedure were chosen based on an extensive literature study (e.g., Florentine & Buus 1984; Glasberg & Moore 1992; Grose & Buss 2007) and considerations made based on pilot studies.

The gap detection test used a three interval three alternative forced choice method. In each trial, the subject was shown three boxes, labeled “Sound 1,” “Sound 2,” and “Sound 3,” referring to the three alternatives, each with an accompanying auditory stimulus. One randomly chosen alternative contained a gap. The subject was asked to select the box with the stimulus containing the gap. Once a selection was made, the test provided visual feedback above the correct box by displaying a smiling or frowning emoticon, according to a correct or incorrect answer, respectively.

GDTs were determined with a two-down/one-up adaptive procedure (2D1U), corresponding to the 70.7% point of the subject’s psychometric function (Levitt 1971). The gap size at the start of the test was 30 msec. After two correct answers, the gap size decreased by a factor of 1.2; after one wrong answer, the gap size increased by a factor of 1.2. The test was terminated after eight reversals, and the GDT was calculated as the geometric mean of the gap sizes at the last five reversals. For each subject, each stimulus, and each level, the GDTs were averaged across the three repetitions. All data were stored in a MATLAB structure array with multiple fields for later analysis.

### Data Analysis

All data were exported from MATLAB to IBM SPSS Statistics 20 for statistical analysis. The Con group is included as a reference group. This means that the results of this group will be shown in figures, but none of the results were used for statistical purposes.

For each stimulus bandwidth, a two-way mixed-model repeated-measures ANOVA with group (Tin versus Notin) as a between-subject factor and level (5, 10, and 25 dB SL) as a within-subject factor was used. The Greenhouse–Geisser correction was used to correct for violations of sphericity.

To investigate a possible relation between tinnitus pitch and gap detection performance, subjects were stratified with respect to their tinnitus pitch. Six subgroups were defined: tinnitus pitch “<4.0 kHz,” “4.0–5.0 kHz,” “5.0–6.3 kHz,” “6.3–8.0 kHz,” “>8.0 kHz,” and “WB,” respectively. Per subgroup, the average GDT was calculated for each of the four stimulus bandwidths, respectively. These averages were computed across stimulus levels. For each tinnitus pitch subgroup, we determined whether the GDT was poorer when the bandwidth of the stimulus overlapped with the matched tinnitus frequency.

## RESULTS

Although the analyses were only performed on the results belonging to the Tin and NoTin groups, the results of the Con group are added to the figures or tables for information purposes.

### Tinnitus Matching

For each subject and all sessions, the matched tinnitus characteristics such as bandwidth, frequency, and sensation level are listed in Table 2. With respect to the matched bandwidth, no large variation across sessions was found. In 17 of the 22 subjects, the bandwidth remained unchanged across the sessions. This table shows that most subjects matched their tinnitus to a tone and only a few subjects to a WB noise. Within subjects, variation in matched tinnitus frequency across sessions is clearly present. In at least 6 of the 22 cases, octave confusions appear (e.g., subjects 4, 5, 6, 7, 15, and 20). Furthermore, a large variability for tinnitus sensation level is displayed.

**TABLE 2.**
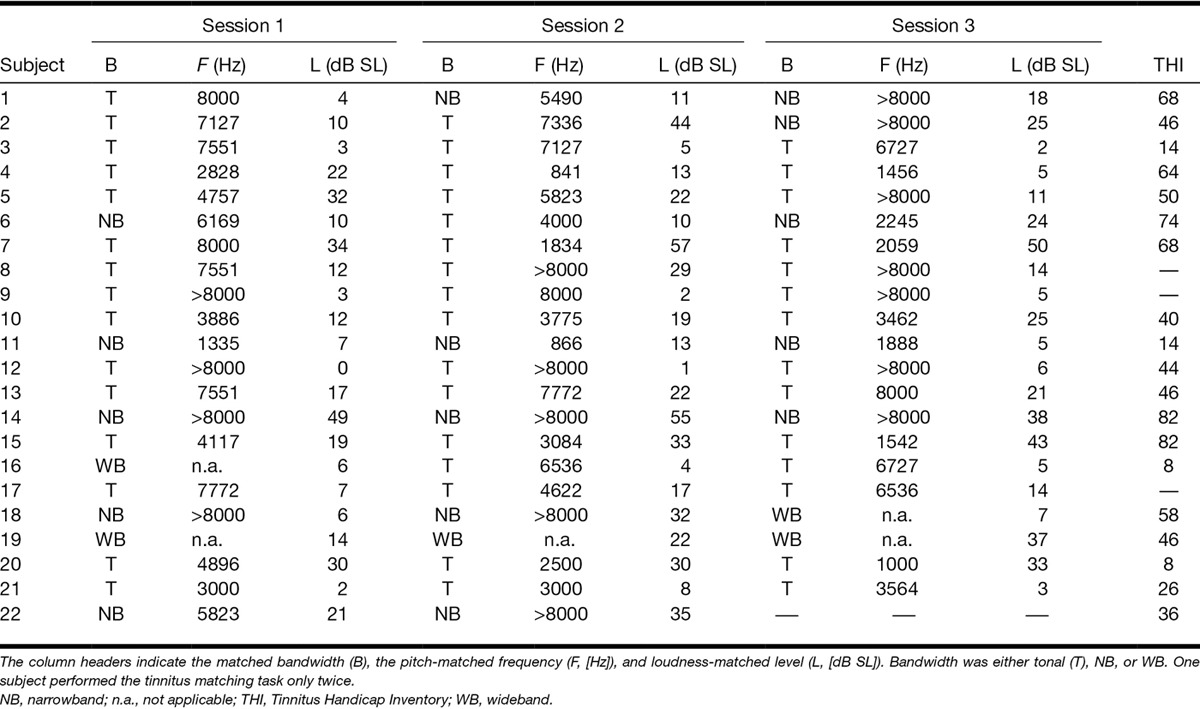
Matched tinnitus frequencies of the tinnitus matching task per tinnitus subject

**Fig. 2.**
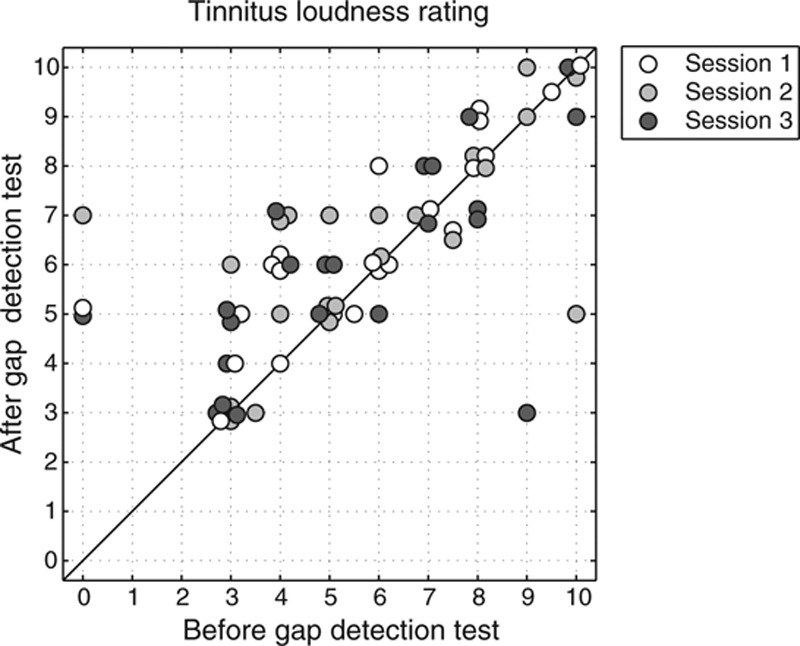
Tinnitus loudness ratings before and after the gap detection test, shown for each Tin subject and each session. The ratings were measured by means of a numeric rating scale from 0 (inaudible) to 10 (tinnitus sounds as loud as imaginable).

The subjectively perceived tinnitus loudness before and after gap detection testing is shown in Figure 2. Results are available for all tinnitus subjects. For one subject, the tinnitus was masked by environmental sounds before testing but was audible after each test session. An overview of the averaged ratings before and after the gap detection test is given in Table 3. In general, the tinnitus loudness was either slightly increased or unchanged during performing the gap detection test.

**TABLE 3.**
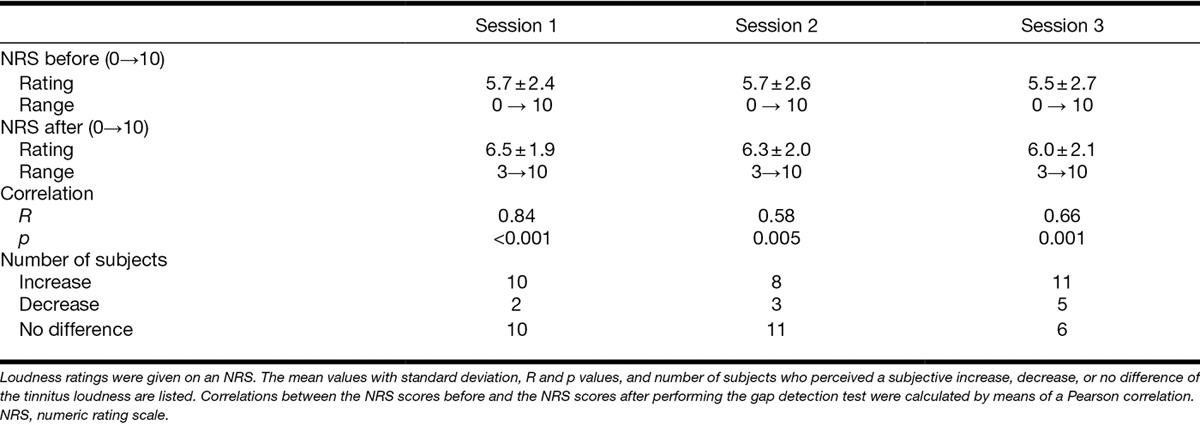
Tinnitus loudness ratings before and after the gap detection test

### Gap Detection

In total, all but three subjects performed each gap detection test three times for each stimulus level and frequency content. For subsequent analyses, the GDTs were averaged across the three measurements. Exceptions were made for two Tin subjects and one NoTin subject, who could not tolerate the loudness of 25-dB SL stimuli and thus did not complete the respective runs.

**Fig. 3.**
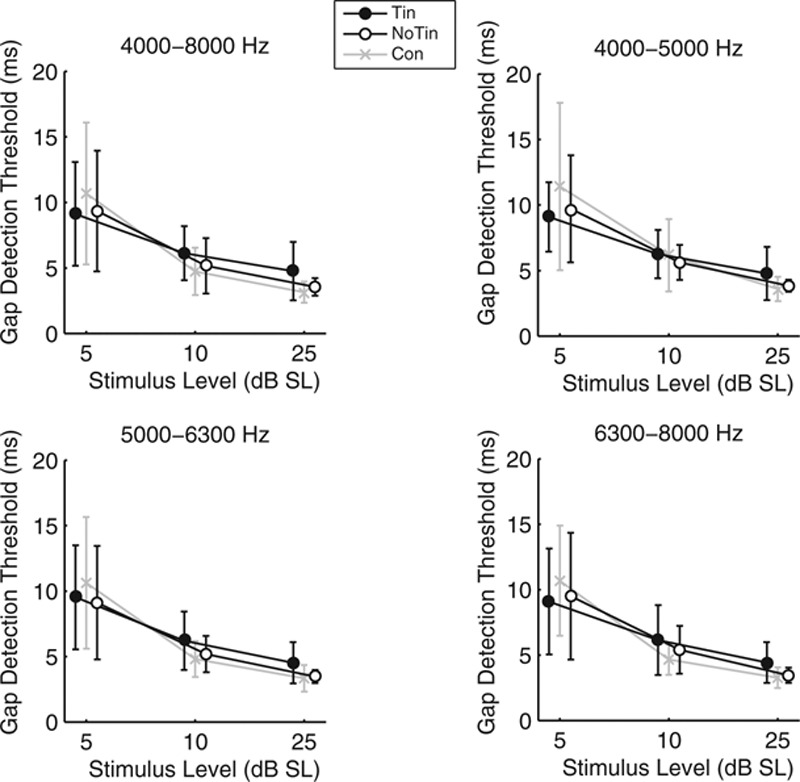
Mean gap detection thresholds of the four test stimuli at three stimulus levels (5, 10, and 25 dB SL) for the Tin group, NoTin group, and Con group. The gap detection thresholds are averaged across sessions. The error bars indicate the group standard deviations around the mean. Con indicates young normal-hearing subjects; NoTin, matched group without tinnitus; Tin, tinnitus.

Figure 3 shows the mean GDTs per group for each of the four test stimuli. All panels show that increasing sensation level yielded better gap detection performance in all groups. Visual inspection of data shows no large differences in GDTs between the three subject groups.

The repeated-measures ANOVA of the GDTs showed no main effect of group for any of the four test stimuli (groups: Tin and NoTin; for each of the four ANOVAs *p* > 0.25). In contrast, a significant main effect of level was found for all stimuli (4000–8000 Hz: *F*(2,40) = 59.86, *p* < 0.001; 4000–5000 Hz: *F*(2,40) = 78.80, *p* < 0.001; 5000–6300 Hz: *F*(2,40) = 56.35, *p* < 0.001; and 6300–8000 Hz: *F*(2,40) = 58.35, *p* < 0.001). No significant interaction effects were found.

**Fig. 4.**
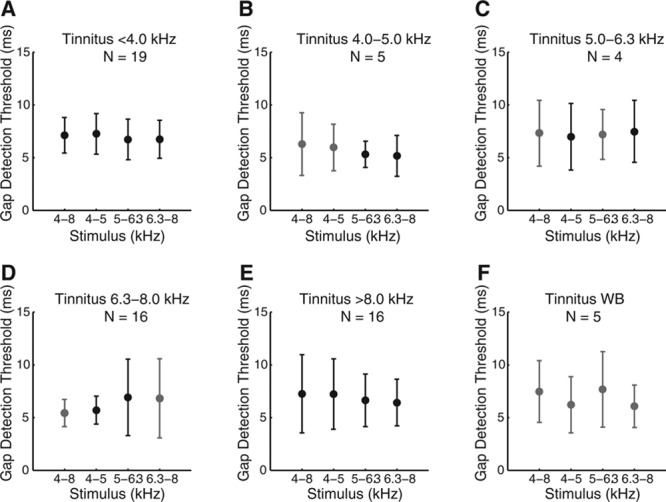
Gap detection thresholds related to perceived tinnitus pitch. Subjects were stratified with respect to the perceived pitch of their tinnitus. A, “<4.0 kHz.” B, “4.0–5.0 kHz.” C, “5.0–6.3 kHz.” D, “6.3–8.0 kHz.” E, “>8.0 kHz.” F, “WB.” In each of these six sets, the gap detection thresholds of 19, 5, 4, 16, 16, and 5 subjects, respectively, were included. Red symbols reflect cases where the frequency band of the stimulus used to assess the gap detection threshold overlapped the perceived tinnitus pitch. The error bars indicate the standard deviations around the mean.

Stratification of the tinnitus subjects with respect to their tinnitus pitch resulted in six subgroups including 19, 5, 4, 16, 16, and 5 subjects, respectively (the tinnitus pitch ranges were “<4.0 kHz,” “4.0–5.0 kHz,” “5.0–6.3 kHz,” “6.3–8.0 kHz,” “>8.0 kHz,” and “WB”). These group sizes reflect a total of 65 measures, corresponding to 21 tinnitus subjects who performed the tinnitus matching task three times and one subject who performed the tinnitus matching task only twice (Table 2). Figure 4 shows the GDTs per subgroup. Visual inspection shows no evidence of a poorer GDT when frequency range of the test stimulus overlapped with the tinnitus pitch.

## DISCUSSION

The main aim of this study was to investigate gap detection performance in patients with tinnitus and to compare this to control subjects, matched in age and hearing loss, but without tinnitus. There were no significant differences in the GDTs of subjects with and without tinnitus. Moreover, there was no relation between the frequency content of the stimulus used to measure the GDT and the tinnitus matching frequency.

The GDT depends on the acoustic parameters of the stimulus in which the gap is to be detected. Concerning stimulus level, it has been shown that GDTs improve with increasing stimulus levels (Fitzgibbons 1983; Moore et al. 1993; Hall & Grose 1997; Horwitz et al. 2011; Hess et al. 2012). In line with the literature, the results of our study also showed a GDT improvement with increasing stimulus level.

The normal GDTs in tinnitus subjects included in this study are consistent with a normal and flawless ability to detect a 50-msec gap (Campolo et al. 2013). Also, they are consistent with the lack of pronounced gap detection deficits in aspirin-induced hearing loss, where the aspirin presumably also induced tinnitus in some subjects (McFadden et al. 1984; note that tinnitus was not explicitly checked). However, in contrast to these results on the conscious perception of a gap, studies that assessed suppression of the startle reflex by a prepulse gap suggest a deficit in gap processing (Fournier & Hébert 2013). In animals, a gap-processing deficit follows after application of conditions that are known to cause tinnitus in humans, such as noise trauma (Turner et al. 2006). In humans, tinnitus is also associated with a deficit in gap processing although deficits were not limited to stimuli with a pitch similar to that of the tinnitus (Fournier & Hébert 2013). Thus, whereas experiments that use the startle reflex show a deficit in detecting a silent gap in animals as well as in humans (Turner et al. 2006; Fournier & Hébert 2013), the psychoacoustic experiments that probe conscious perception of a gap do not show such a deficit (Campolo et al. 2013; this work).

How can it be explained that the startle reflex appears to be impaired in human patients with tinnitus whereas conscious perception of a gap measured psychophysically is normal? Possibly the difference is related to differences in the neural circuits that are involved in the startle reflex and the conscious psychoacoustic task. The startle reflex is based on a subcortical pathway (Davis et al. 1982; Lee et al. 1996). In contrast, the psychoacoustic task presumably includes the auditory and motor cortex (Zschorlich & Köhling 2013), in addition to the subcortical auditory structures. The fact that an impairment is only found in the startle experiment suggests that the startle paradigm specifically probes a subcortical deficit. In other words, the startle paradigms may probe preattentive mechanisms (Campolo et al. 2013), whereas the conscious psychoacoustic tasks clearly involve attention (Cromwell et al. 2008).

More recent experiments in animals cast some doubt on the applicability of the gap detection startle paradigm to detect tinnitus. In a condition in which Hickox and Liberman (2014) suggested to maybe cause tinnitus, only marginal deficits in gap detection were observed. Hence, the startle paradigm may be inadequate to detect tinnitus in these cases. This is a conclusion that obviously depends on the assumption that animals actually had tinnitus. Another paper described changes in the startle response amplitude in conditions where the animals were not assumed to have tinnitus (Lobarinas et al. 2013). For example, induced conductive hearing loss was assumed not to have caused tinnitus, but it produced startle changes that may be interpreted as tinnitus in the experimental animals. However, conductive hearing loss may also cause tinnitus (Mills & Cherry 1984). Consequently, using conductive hearing loss as a control for no-tinnitus cases may not be adequate (Heeringa et al. 2014). Thus, although these experiments (Hickox & Liberman 2014; Lobarinas et al. 2013) cast doubt on the applicability of the gap detection startle paradigm, the validation of behavioral paradigms in animals depends on assumptions the experimenter makes on the presence of tinnitus. Nevertheless, taking together the results of the animal and human studies, the usefulness of gap detection methods in animals and humans may be questioned.

An initial motivation for conducting the present study was to find support for gap detection paradigms used in animal tinnitus studies. The lack of gap detection deficits does not provide support for these methods. The finding that gaps were easily detected by human subjects with tinnitus may suggest that the gap detection task is too simple in its current form. Possibly, cortical processing in humans compensates for minor subcortical deficits. In other words, although minor subcortical deficits associated with tinnitus may cause abnormal inhibition of ASR (Fournier & Hébert 2013), these do not lead to abnormalities in the detection of gaps as used in the current paper. Thus, in humans, it is possible that tinnitus does not fill the gap sufficiently to disrupt gap detection. Regardless of the exact mechanisms underlying the lack of a tinnitus effect on gap detection, the present results indicate that a simple gap detection is not yet a suitable clinical tool to identify tinnitus.

## ACKNOWLEDGMENTS

The study is part of the research program of our department, Healthy Aging and Communication. The authors thank Renée Koolschijn, Korien Leemhuis, Chloë De Schepper, Fabian van Luyn, Esmée van der Veen, Floor Burgerhof, and Etienne Gaudrain for their specific contribution to this work.

This research was funded by the Action on Hearing Loss, the Dorhout Mees Family Foundation, the Heinsius Houbolt Foundation, The Netherlands Organisation for Scientific Research (NWO), and the University of Groningen (Rosalind Franklin Fellowship to D. B.).
